# Valuing embodiment: insights from dance practice among people living with dementia

**DOI:** 10.3389/fneur.2023.1174157

**Published:** 2023-06-05

**Authors:** Magda Kaczmarska

**Affiliations:** ^1^Global Brain Health Institute, San Francisco, CA, United States; ^2^Foundation Dementia Action Alliance Poland, Sopot, Poland

**Keywords:** dance, embodiment, dementia, neuroscience, movement, brain health

## Abstract

There is a growing appreciation for the ability of person-centered arts-based approaches to extend multiple domains of brain health of people living with dementia. Dance is a multi-modal artistic engagement which has positive impacts on cognition, mobility and the emotional and social aspects of brain health. Although research into multiple domains of brain health among older adults and people living with dementia is promising, several gaps remain, specifically in understanding the benefits of co-creative and improvisational dance practices. Collaborative research between dancers, researchers, people living with dementia and care partners is needed to design and evaluate future research on dance and to determine relevance and usability. Furthermore, the respective praxes and experience of researchers, dance artists and people living with dementia contribute distinctly and uniquely to the identification and the assignment of value to dance in the context of the lives of people living with dementia. In this manuscript the author, a community-based dance artist, creative aging advocate and Atlantic Fellow for Equity in Brain Health, discusses current challenges and gaps in the understanding of the value of dance for and with people living with dementia and how transdisciplinary collaboration between neuroscientists, dance artists and people living with dementia can advance collective comprehension and implementation of dance practice.

## Introduction

Dementia is an umbrella term for conditions that affect cognitive function, thinking and social abilities of individuals to the extent that interfere in their daily lives (1). Currently people living with dementia (PLWD) constitute 50 million globally, with projections reaching 153 million by 2050 (2). PLWD experience stigma (3) and multi-layered marginalization that is compounded by the detrimental effects of a progressive chronic illness which can impact affective states (4), mobility and spatial relationships (5, 6), and ability to communicate or follow conversations (7) all of which can hinder meaningful engagement and connection. As a result, PLWD inevitably experience shifts in their modes and capacities for expression which influence their ability to express and feel connected to the communities around them. Reportedly, PLWD and their care partners often experience a reduction in size of their social networks and loss of connection with others as the disease progresses (8). The value and position of the arts as a vehicle for social justice to advance a culture shift toward inclusion, creativity and respect of PLWD at every level of society is increasingly recognized (9), whereas the need for programs that engage and involve people living with dementia as co-creators is apparent (10, 11).

Dance is an art form increasingly recognized for its role to promote brain health (a life-long, multidimensional and dynamic state consisting of cognitive, emotional and motor domains) (12) and wellbeing (13–16) in part through community engagement coupled with physical fitness. Dance, for people living with dementia, centers dignity and meaning and “takes on even greater significance given that corporeality becomes the primary means of engaging with the world and with other.” As Kontos states, “Dance provides a unique medium for non-verbal communication, affect and reciprocal engagement which profoundly enables the relational citizenship of persons living with dementia” (17). Nonetheless, compared to other creative practices, the correlation of dance on the physical, cognitive and psychosocial wellbeing of PLWD is under-represented in neuroscience research and understanding of certain types of dance, such as co-creative dance, among PLWD is entirely lacking.

Neuroscientists define dance as a multi-modal activity that couples cognitive tasks with aerobic exercise and social engagement (18). Warburton defines dance as a conscious event, which deploys implicit (procedural) and explicit (declarative) knowledge presenting both neurobiological and phenomenological features (19). Within academic neuroscience research, contradictory, incomplete or over-generalizing representations of dance are common (19). The impact of dance interventions is not reported in a standardized way rendering replicability, relatability to practice and correlation between results of multiple studies challenging (20–25). Often, results from interventions of diverse dance forms are reported together without comprehensive discussion or inference of the findings on their practice (26–29). To map the complex interconnected elements of diverse types of dance practice on multiple domains of well-being, we need trans-disciplinary teams collaborating to design studies, evaluate results and determine implementation. Dance artists bring a specialized skillset to their praxis which is honed through experiential research co-created with their communities of practice. Neuroscientists can help clarify and validate what dancers witness in practice, in turn expanding the resources they have and backing them with evidence. In response, dancers and neuroscientists can work in a mutually beneficial feedback loop of inquiry and application founded in multi-faceted knowledge and evidence. In this manuscript, the author, a community-based dance artist, creative aging advocate and Atlantic Fellow for Equity in Brain Health, discusses current challenges and gaps in the understanding of the value of dance for and with PLWD and how transdisciplinary collaboration between neuroscientists dance artists and PLWD can advance collective comprehension and implementation of dance practice.

## Let’s get physical: dance, aerobic activity, and the brain

Research has long recognized the positive connection between aerobic exercise and heart and brain health (30). Aerobic exercise increases cerebral oxygenation and blood flow and promotes angiogenesis within the brain in childhood, adulthood and elderhood (31–33). A pooled cohort study evaluating the link between leisure dancing and cardiovascular risk when compared to walking found that moderate-intensity dance activity was correlated to lower cardiovascular disease and mortality over a moderate aerobic-intensity walking (34). Researchers posited this increased protective factor from moderate-intensity dance may be due to higher-intensity bouts within dance. In fact, sports medicine researchers recognize high-intensity interval training as superior in promoting cardiovascular fitness than moderate-intensity continuous aerobic activities (35).

Physical activity in later life was found to increase levels of synaptic proteins in a global (not limited to one region of the brain) and time-dependent fashion (people active up to 0–2 years to end of life have bigger effect). Researchers hypothesize that one reason for this is linked to the cardiovascular connection to brain health (36). As synaptic integrity promotes connectivity in the brain and is seen as a biologic precursor of cognition, this research posits profound implications for physical activity started in later life. When analyzing brain tissue, Casaletto et al. found physical activity provides a powerful neuroprotective factor even in light of existing neuropathologies (36). Although Casaletto’s research did not specify a type of physical activity, other research has illustrated how aerobic activity in the form of dance not only supports synaptic integrity but engages circuits that connect regions of the brain (37).

Growing evidence points to forms of dementia like Alzheimer’s disease as being related to brain connectivity issues that hinder communication between areas of the brain (38–40). The medial temporal lobe is one of the first networks in the brain to be impacted by neurodegenerative conditions like Alzheimer’s disease (41). Sinha et al. tested cognitive flexibility and measured the functional activity within the medial temporal lobes of participants (ages 65+) engaged in moderate aerobic dance and analyzed participants’ ability to acquire new information and correlate it to previous knowledge. Those who engaged in the dance program had higher neural flexibility within the medial temporal lobe network which also reflected in cognitive tests and learning new tasks, specifically by learning a new idea and applying it in another context. As a conclusion, the team asserted aerobic dance exerts a rehabilitative and protective effect on medial temporal lobe function (37).

Aerobic activity promotes neurogenesis. Rehfeld et al. matched dance (aerobic dance phrases of increasingly more complex and varying choreography) and conventional fitness training (bicycle ergometer, resistance training and flexibility training) for aerobic exertion. Participants (ages 60+) who joined in the dance program showed a volumetric increase in the medial temporal lobe as well as higher levels of brain derived neurotrophic factor (BDNF) in their plasma (42). BDNF supports synaptic strength as well as neuronal plasticity, specifically through increased neurogenesis by promoting neuronal cell survival and proliferation, and is positively correlated to increases in cognition (43). Rehfeld et al. posit BDNF increase after dance over repetitive fitness training is due to dance coupling aerobic activity with novelty and challenge (42).

## Steppin’ out: gait as a prodromal marker for brain health

Verghese et al. investigate gait as a prodromal marker for dementia (44). In a study of older adult long-term leisure partner dancers, they observed that compared to non-dancers, they exhibited overall greater balance, which included better gait. However, the dancers did not have greater strength than non-dancers (44). As both sarcopenia and decreased gait are correlated to increased risk of developing dementia (45, 46), this illustrates how dance, when coupled with additional resistance training or specific targeted activities that build muscle strength and endurance could provide a holistic support to functional mobility while off-setting detrimental brain health in advancing age.

## I want to dance with somebody: dance enhances mood and social connection

Dance engagement among people living with dementia in residential care settings increases mood and social interaction (47). Engagement in 12 weeks of dance at a residential nursing home reduced the need for depression medication among older adult participants (including people with diagnoses of dementia) (48). Dance-based interventions are significantly beneficial to persons with MCI and dementia in decreasing depression compared with controls, with similar effects in both hospital and community settings (49). This impact on mood may occur due to the aerobic engagement of dance which increases blood flow to the brain (50) as well as from intrinsic reward, which results in neural synchrony, enhanced interpersonal coordination, and an avenue for pro-social behaviors (51).

Dance promotes pro-social behaviors. Dancers and musicians both exhibit higher levels of measures of empathy than controls and consistently higher insular connectivity (52). Researchers posit that this heightened empathetic and insular sensitivity can be attributed to both art forms requiring frequent feedback between internal and external cues. Mirroring is a common practice utilized in dance to promote heightened relational awareness. Engaging in facial mirroring synchronously activates the same areas of the brain, namely, areas that are involved in social connection: the prefrontal cortex, insula and temporo-parietal junction (53). Researchers posit that engaging in physical mirroring, amplifies the practice of empathy (54). Although a definitive theoretical framework explaining mechanisms of empathy in dance mirroring is unclear (55), what is understood is the sensorimotor correlation of empathy and pain (56), which is related to mirror systems (57). Neural synchrony of doctors and patients involved in mirroring resulted in analgesic effects for the patients (53). The extent to which this experience correlates to dance mirroring remains to be discovered, but upcoming research from Emily Cross and team might illuminate this (58).

Dance, specifically co-creative dance improvisation, can be seen as a form of awe, by cultivating an intention and perspective of curiosity, reciprocity, and cooperation. “By shifting attention away from the self and onto the outside world, awe diminishes feelings of self-importance and makes people feel smaller, yet more connected, to a larger community and purpose” (59). Keltner speaks of the ability of engaging in the practice of awe to place individuals neurophysiologically “in sync” with one another, in part through the phenomenon of “Collective Effervescence” or “just moving together, feeling exalted, bubbling, being ecstatic” (60). Dance promotes novelty and a sense of awe within the framework of the quotidian. In application with older adults or PLWD who may find leaving their residence difficult, the value of an approach that cultivates enrichment, awe, and novelty within the ordinary should not be overlooked. Cumulatively, the application of awe and novelty, through the practice of shared dance, has the potential to induce small positive stress resulting in neuroplasticity, while promoting a sense of well-being and belonging. More research is needed to make a direct correlation between mirroring movement, co-creative dance engagement and regions in the brain indicated in social connection, pro-sociality and empathy, but these studies imply promising connection.

## Just my imagination? Dance, creativity and improvisation

Neuroplasticity refers to the brain’s ability to modify, change, and adapt both structure and function throughout life and in response to experience (61, 62). whereas access to new physical spaces, learning new skills, training memory, playing games, participating in activities associated with spatial learning and motor coordination (i.e., sports, artistic and creative activities,) and a developed social life all constitute an enriched environment (EE) (63). EE benefits global brain health domains resulting in neuroplasticity (64). Per Kempermann, higher physical activity leads to increased proliferation of neurons, synaptogenesis, and more dendritic complexity, while enrichment leads to higher rates of survival of newly formed neuronal cells and recruitment into lasting functional integration. This long-lasting functional integration can be identified as cognitive reserve (65). Robertson argues that the type of positive stress that we experience when we introduce challenge, novelty, and the element of surprise leads to lifelong cognitive reserve (66). Adding an additional task or challenge to an already established task, can add positive stress resulting in increasing noradrenaline, which, can in turn can lead to improved attention and, when sustained, ultimately result in neuronal plasticity (65).

Improvisation in dance is a process-based form of movement inquiry that occurs either individually or in groups and cultivates a “practice of dwelling in possibility” (67). Dance improvisation is experiential by definition and “generates understanding about the phenomena of being through ‘presencing’ a polyattentive body, conversing with ambiguity, uncertainty, potentiality, and choice” (68). Through this lens, dance improvisation can enrich the environment in a way that cultivates opportunity for challenge and positive stress, with beneficial potential for neuroplasticity and focus.

EE fosters creativity, a multifaceted phenomenon, that uses imagination or original ideas to achieve valued goals (69). One of the dominant ways of evaluating and looking at creativity in neuroscience is by exploring divergent versus convergent thinking. Divergent thinking, which is often classified as playful, self-aware internal rumination, engages the default mode network (DMN), which, among other areas, depend on activation of the medial prefrontal cortex for evaluation of internal stimuli as a core component of the self-awareness network. Convergent thinking depends on the lateral prefrontal and parietal cortices, which are core components of the executive control network (ECN) that supports purposeful use of symbols and intentional inhibition of externally triggered impulses (70). Neuroscience frequently represents divergent and convergent thinking as antagonistic.

Research from Beaty et al. illustrate how among “highly creative individuals” these two ways of thinking and their related brain networks work in concert with one another (71). “Highly creative” individuals exhibit a higher connectivity when performing a creative thinking task between the DMN and the inferior prefrontal cortex, a part of the brain related to the ECN (71) Research from Dr. Charles Limb illuminates potential neurophysiological mechanisms that corroborate this phenomenon through examination of musical jazz solo vs. partners improvisation (72, 73). In comparison to memorized, non-improvised exchange, partner improvised musical exchange was characterized by functional connectivity in the inferior frontal gyrus, the parts of the brain that regulate executive control areas that are linked to cognitive and emotional processing, inhibition, and attention (72). In contrast, solo jazz improvisation resulted in the down regulation of the inhibition network and increased activation in the sensory cortex (73) which correlates to what is recognized during activation of the DMN linked to self-expression and rumination (73). In comparison to solo musical improvisation, there is greater expectation during a musical conversation for relationship and continuity thus increased self-monitoring coupled with rumination is required (72). This data correlates to the research by Beaty and team who recognized that among highly creative individuals, default network engagement was coupled with functional connectivity in the inferior frontal gyrus, an area of the brain involved in inhibition, direction, and executive control.

This research suggests that an improvisational approach invites proficiency in content but trains a flexibility between the global and the local and could offer superior benefits in neuronal engagement and plasticity. Dance improvisation adds the benefits of improvisation to the other emotional, social, physical, and cognitive benefits of dance engagement. Solo dance improvisation, although sometimes responsive to external stimuli is characterized by drawing internally in a ruminative fashion on memory of movement and sensory stimuli with a lack of inhibition of content development. Group dance improvisation and co-creative or collaborative dance making, requires a combination of “in the moment” selective content creation responsive to both external and internal cues, similarly drawing on former embodied knowledge. In this light, a model of both dance and music improvisation might be seen as (1) tapping into a library of accumulated cerebral and corporeal information and reconnecting it in novel ways, (2) training a practice of flexibility and connectivity between areas of the brain, and (3) supporting ease with uncertainty. This is beneficial for supporting brain plasticity and adaptability and has potentially powerful protective brain health implications for diverse ages and abilities. Although research into the specific mechanisms taking place during solo and group dance improvisation is lacking, the similarity in the practice of dance and musical improvisation invites more inquiry into the extent of the neurological similarities and differences between them.

For people living with dementia and care partners, improvisation cultivates engagement (74, 75), but a need persists to understand which brain networks are engaged when they participate in dance improvisation. Future studies should explore functional connectivity among older adults and people living with dementia when engaging in solo and group dance improvisation to establish the extent to which dance improvisation relates to the above noted impact of musical improvisation.

## Value of dance for and with people living with dementia

The above review of literature illustrates the benefits of dance for people living with dementia but the value of dance goes beyond what can be quantified through empirical research. Reduction of dance to measurable physiological changes fails to capture its full value to and for people living with dementia. As illustrated by Kontos, adaptation of dance solely for instrumental and therapeutic purposes, impoverishes the understanding of the value of dance for people living with dementia and fails to recognize the ability of dance to promote self-hood, support embodied forms of communication and meaningful collective engagement (17).

A case study of a co-creative dance program for people living with dementia (Supplementary Box 1) emphasizes existing modes of communication by recognizing and coupling verbal and embodied forms of expression. The resulting collaborative dance practices amplify and extend agency, personhood and meaningful connection while building new spaces of belonging and community.

Stories in the Moment™ is a co-creative dance, movement, and storytelling program for people living with dementia and care partners that was developed by the author (76). The intention behind Stories in the Moment™ is to amplify the creative voice of people living with dementia while supporting them and care partners in extending their resources for meaningful communication through individual and collective dance. The class structure balances dance activities led by the facilitator with individual and collective dance improvisation to result in co-created dance stories and miniature dance performances. Activities in the class apply existing evidence-informed benefits of dance on multiple domains of brain health of older adults and people living with dementia but see these as secondary to the primary intention of the program which is to utilize co-creative and improvisation dance to promote agency, personhood and relationality. Frameworks and tools adapted from group dance improvisation support engagement by heightening the awareness of participants’ “kinesthetic sense” while encouraging modes of communication most comfortable and accessible to them.

## Discussion

Dance is an aesthetic art form that couples beneficial engagement in multiple domains of brain health with relationality and meaning. As we consider the value of dance on health and wellbeing, we need to ensure we include the contributions of the artists and the participants (people living with dementia and care partners) who will be integral to the implementation and growth of this work alongside those of the clinicians, researchers, and policymakers.

Currently, there are gaps in knowledge, and dance continues to be under- and misrepresented in neuroscience research. Without this knowledge, policymakers lack the evidence-base behind which to sponsor and create recommendations for use and clinicians are reticent to prescribe or promote dance practice for their patients. More research is needed to build on preliminary data that is offering promising benefits of diverse forms of dance on multi-modal domains of brain health and well-being. This new research must happen with artists and people living with dementia at the table.

In order to ensure that the knowledge of the lived experience experts (people living with dementia), the applied practice experts (artists) as well as the neuroscientists is acknowledged and leveraged with existing knowledge from empirical, qualitative and experiential research, a horizontal, collaborative and equitable approach, rather than a hierarchical approach is needed (75). If we do not change the systems in which we identify impact and value by expanding and normalizing trans-disciplinary collaborations between dance, neuroscience and the communities they serve, we risk losing the spirit of why these programs actually work – their creativity, their flexibility, their reciprocity, their humanity. At its essence, approaching collaboration from the horizontal allows “for multiple perspectives and recogniz[ing] that making distinctions is a creative act and worth doing in order to understand the nuances of our efforts… [within this approach] many ideas can coexist” (77).

Emily Cross posits what might constitute this horizontal collaborative process between artists and neuroscientists by amplifying a need for a bi-directional listening and cultivating collaborations with the arts that include a two-way communication from the ground up: “arts research is hugely vital in understanding who we are as a human culture and where we come from and where we are going, and science does not really give us that. The value of arts research for arts within itself is tremendous and it does not need science to legitimize itself” (78). In adopting a collective practice of awe and improvisation that invoke deep listening, witnessing and mutual respect, we can build authentically collaborative and mutually beneficial relationships between the arts, research, and the communities we serve.

## Author’s note

Magda Kaczmarska, MFA is a dance artist, creative aging thought leader and Atlantic Fellow for Equity in Brain Health. She is the Vice President of Foundation Dementia Action Alliance Poland and serves on the board of the Dementia Action Alliance (United States). Kaczmarska founded DanceStream Projects, a creative collective based in New York City, that cultivates transdisciplinary partnerships to provide direct ally-ship and empowerment to communities by bridging arts and health and centering dance as a catalyst for systems change. Kaczmarska mentors future leaders in the creative and health sectors through regular partnership at the Fordham Ailey School of Dance in New York City and the Arts in Medicine Fellowship in Lagos, Nigeria. She served on the National Dance Education Organization (NDEO) Dance and Disability Task Force until 2022 and continues to serve as a representative to the UN with Generations United and is on the executive committee of the UN NGO Committee on Ageing.

## Data availability statement

The original contributions presented in the study are included in the article/Supplementary material, further inquiries can be directed to the corresponding author.

## Author contributions

The author confirms being the sole contributor of this work and has approved it for publication.

## Conflict of interest

The author declares that the research was conducted in the absence of any commercial or financial relationships that could be construed as a potential conflict of interest.

## Publisher’s note

All claims expressed in this article are solely those of the authors and do not necessarily represent those of their affiliated organizations, or those of the publisher, the editors and the reviewers. Any product that may be evaluated in this article, or claim that may be made by its manufacturer, is not guaranteed or endorsed by the publisher.

## References

[1] World Health Organization. Global Action Plan on the Public Health Response to Dementia 2017–2025. Geneva: World Health Organization (2017).

[2] GBD 2019 Dementia Forecasting Collaborators. Estimation of the global prevalence of dementia in 2019 and forecasted prevalence in 2050: an analysis for the global burden of disease study 2019. Lancet Public Health. (2022) 7:e105–25. doi: 10.1016/S2468-2667(21)00249-8 34998485 PMC8810394

[3] Low LF Purwaningrum F. Negative stereotypes, fear and social distance: a systematic review of depictions of dementia in popular culture in the context of stigma. BMC Geriatr. (2020) 20:477. doi: 10.1186/s12877-020-01754-x, PMID: 33203379 PMC7670593

[4] Cerejeira J Lagarto L Mukaetova-Ladinska EB. Behavioral and psychological symptoms of dementia. Front Neurol. (2012) 3:73. doi: 10.3389/fneur.2012.00073 22586419 PMC3345875

[5] Kueper JK Speechley M Lingum NR Montero-Odasso M. Motor function and incident dementia: a systematic review and meta-analysis. Age Ageing. (2017) 46:729–38. doi: 10.1093/ageing/afx084, PMID: 28541374

[6] Possin KL. Visual spatial cognition in neurodegenerative disease. Neurocase. (2010) 16:466–87. doi: 10.1080/13554791003730600, PMID: 20526954 PMC3028935

[7] Banovic S Zunic LJ Sinanovic O. Communication difficulties as a result of dementia. Mater Sociomed. (2018) 30:221–4. doi: 10.5455/msm.2018.30.221-224, PMID: 30515063 PMC6195406

[8] Balouch S Rifaat E Chen HL Tabet N. Social networks and loneliness in people with Alzheimer’s dementia. Int J Geriatr Psychiatry. (2019) 34:666–73. doi: 10.1002/gps.5065, PMID: 30706526

[9] Kontos P Radnofsky ML Fehr P Belleville MR Bottenberg F Fridley M. Separate and unequal: a time to reimagine dementia. J Alzheimers Dis. (2021) 80:1395–9. doi: 10.3233/JAD-210057, PMID: 33646169

[10] Ericsson I Kjellström S Hellström I. Creating relationships with persons with moderate to severe dementia. Dementia. (2013) 12:63–79. doi: 10.1177/1471301211418161, PMID: 24336663 PMC4268479

[11] Love K Femia E. Helping individuals with dementia live more fully through person-centered practices. J Gerontol Nurs. (2015) 41:9–14. doi: 10.3928/00989134-20151015-02, PMID: 26505243

[12] Chen Y Demnitz N Yamamoto S Yaffe K Lawlor B Leroi I. Defining brain health: a concept analysis. Int J Geriatr Psychiatry. (2021) 37:1–13. doi: 10.1002/gps.5564 34131954

[13] Guss-West C Jenkins E. Introducing “Dance for Health.” International Association for Dance Medicine and Science; (2019). Available at: https://iadms.org/resources/blog/posts/2019/february/introducing-dance-for-health/ (Accessed February 4, 2023).

[14] Harrison EA Lord LM Asongwed E Jackson P Johnson-Largent T Jean Baptiste AM. Perceptions, opinions, beliefs, and attitudes about physical activity and exercise in Urban-community-residing older adults. J Prim Care Community Health. (2020) 11:215013272092413–7. doi: 10.1177/2150132720924137 PMC7263106 32468912

[15] Verghese J Lipton RB Katz MJ Hall CB Derby CA Kuslansky G. Leisure activities and the risk of dementia in the elderly. N Engl J Med. (2003) 348:2508–16. doi: 10.1056/NEJMoa022252 12815136

[16] Chan JSY Wu J Deng K Yan JH. The effectiveness of dance interventions on cognition in patients with mild cognitive impairment: a meta-analysis of randomized controlled trials. Neurosci Biobehav Rev. (2020) 118:80–8. doi: 10.1016/j.neubiorev.2020.07.017, PMID: 32687886

[17] Kontos P Grigorovich A. Integrating citizenship, embodiment, and relationality: towards a reconceptualization of dance and dementia in long-term care. J Law Med Ethics. (2018) 46:717–23. doi: 10.1177/1073110518804233, PMID: 30336101

[18] Karpati FJ Giacosa C Foster NE Penhune VB Hyde KL. Dance and the brain: a review. Ann N Y Acad Sci. (2015) 1337:140–6. doi: 10.1111/nyas.12632 25773628

[19] Warburton E. Of meanings and movements: re-languaging embodiment in dance phenomenology and cognition. Dance Res J. (2011) 43:65–84. doi: 10.1017/S0149767711000064

[20] Doi T Verghese J Makizako H Tsutsumimoto K Hotta R Nakakubo S. Effects of cognitive leisure activity on cognition in mild cognitive impairment: results of a randomized controlled trial. J Am Med Dir Assoc. (2017) 18:686–91. doi: 10.1016/j.jamda.2017.02.013 28396179

[21] Qi M Zhu Y Zhang L Wu T Wang J. The effect of aerobic dance intervention on brain spontaneous activity in older adults with mild cognitive impairment: a resting-state functional MRI study. Exp Ther Med. (2019) 17:715–22. doi: 10.3892/etm.2018.7006, PMID: 30651855 PMC6307442

[22] Burzynska AZ Jiao Y Knecht AM Fanning J Awick EA Chen T. White matter integrity declined over 6-months, but dance intervention improved integrity of the fornix of older adults. Front Aging Neurosci. (2017) 9:59. doi: 10.3389/fnagi.2017.00059 28360853 PMC5352690

[23] Hackney ME Earhart GM. Effects of dance on movement control in Parkinson’s disease: a comparison of argentine tango and American ballroom. J Rehabil Med. (2009) 41:475–81. doi: 10.2340/16501977-0362, PMID: 19479161 PMC2688709

[24] Niemann C Godde B Voelcker-Rehage C. Senior dance experience, cognitive performance, and brain volume in older women. Neural Plast. (2016) 2016:9837321–10. doi: 10.1155/2016/9837321, PMID: 27738528 PMC5055974

[25] Kropacova S Mitterova K Klobusiakova P Brabenec L Anderkova L Nemcova-Elfmarkova N. Cognitive effects of dance-movement intervention in a mixed group of seniors are not dependent on hippocampal atrophy. J Neural Transm (Vienna). (2019) 126:1455–63. doi: 10.1007/s00702-019-02068-y, PMID: 31452049

[26] Wu CC Xiong HY Zheng JJ Wang XQ. Dance movement therapy for neurodegenerative diseases: a systematic review. Front Aging Neurosci. (2022) 14:975711. doi: 10.3389/fnagi.2022.975711, PMID: 36004000 PMC9394857

[27] Hewston P Kennedy CC Borhan S Merom D Santaguida P Ioannidis G. Effects of dance on cognitive function in older adults: a systematic review and meta-analysis. Age Ageing. (2021) 50:1084–92. doi: 10.1093/ageing/afaa270, PMID: 33338209

[28] Wu VX Chi Y Lee JK Goh HS Chen DYM Haugan G. The effect of dance interventions on cognition, neuroplasticity, physical function, depression, and quality of life for older adults with mild cognitive impairment: a systematic review and meta-analysis. Int J Nurs Stud. (2021) 122:104025. doi: 10.1016/j.ijnurstu.2021.104025, PMID: 34298320

[29] Meng X Li G Jia Y Liu Y Shang B Liu P. Effects of dance intervention on global cognition, executive function and memory of older adults: a meta-analysis and systematic review. Aging Clin Exp Res. (2020) 32:7–19. doi: 10.1007/s40520-019-01159-w, PMID: 30982217

[30] Hillman CH Erickson KI Kramer AF. Be smart, exercise your heart: exercise effects on brain and cognition. Nat. Rev. Neurosci. (2008) 9:58–65. doi: 10.1038/nrn2298 18094706

[31] Chaddock-Heyman L Erickson KI Chappell MA Johnson CL Kienzler C Knecht A. Aerobic fitness is associated with greater hippocampal cerebral blood flow in children. Dev Cogn Neurosci. (2016) 20:52–8. doi: 10.1016/j.dcn.2016.07.001, PMID: 27419884 PMC6987716

[32] Kleinloog JPD Mensink RP Ivanov D Adam JJ Uludağ K Joris PJ. Aerobic exercise training improves cerebral blood flow and executive function: a randomized, controlled cross-over trial in sedentary older men. Front Aging Neurosci. (2019) 11:333. doi: 10.3389/fnagi.2019.00333, PMID: 31866855 PMC6904365

[33] Bliss ES Wong RH Howe PR Mills DE. Benefits of exercise training on cerebrovascular and cognitive function in ageing. J Cereb Blood Flow Metab. (2021) 41:447–70. doi: 10.1177/0271678X20957807, PMID: 32954902 PMC7907999

[34] Merom D Ding D Stamatakis E. Dancing participation and cardiovascular disease mortality: a pooled analysis of 11 population-based British cohorts. Am J Prev Med. (2016) 50:756–60. doi: 10.1016/j.amepre.2016.01.004, PMID: 26944521

[35] Hannan AL Hing W Simas V Climstein M Coombes JS Jayasinghe R. High-intensity interval training versus moderate-intensity continuous training within cardiac rehabilitation: a systematic review and meta-analysis. Open Access J Sports Med. (2018) 9:1–17. doi: 10.2147/OAJSM.S150596, PMID: 29416382 PMC5790162

[36] Casaletto K Ramos-Miguel A VandeBunte A Memel M Buchman A Bennett D. Late-life physical activity relates to brain tissue synaptic integrity markers in older adults. Alzheimers Dement. (2022) 18:2023–35. doi: 10.1002/alz.12530, PMID: 34994517 PMC9259753

[37] Sinha N Berg CN Yassa MA Gluck MA. Increased dynamic flexibility in the medial temporal lobe network following an exercise intervention mediates generalization of prior learning. Neurobiol Learn Mem. (2021) 177:107340. doi: 10.1016/j.nlm.2020.107340, PMID: 33186745 PMC7861122

[38] Palop JJ Mucke L. Amyloid-beta-induced neuronal dysfunction in Alzheimer’s disease: from synapses toward neural networks. Nat Neurosci. (2010) 13:812–8. doi: 10.1038/nn.2583, PMID: 20581818 PMC3072750

[39] Busche MA Konnerth A. Impairments of neural circuit function in Alzheimer’s disease. Philos Trans R Soc Lond Ser B Biol Sci. (2016) 371:20150429. doi: 10.1098/rstb.2015.0429, PMID: 27377723 PMC4938029

[40] Kim S Nam Y Kim HS Jung H Jeon SG Hong SB. Alteration of neural pathways and its implications in Alzheimer’s disease. Biomedicine. (2022) 10:845. doi: 10.3390/biomedicines10040845, PMID: 35453595 PMC9025507

[41] Raslau FD Mark IT Klein AP Ulmer JL Mathews V Mark LP. Memory part 2: the role of the medial temporal lobe. AJNR Am J Neuroradiol. (2015) 36:846–9. doi: 10.3174/ajnr.A4169, PMID: 25414002 PMC7990589

[42] Rehfeld K Lüders A Hökelmann A Lessmann V Kaufmann J Brigadski T. Dance training is superior to repetitive physical exercise in inducing brain plasticity in the elderly. PLoS One. (2018) 13:e0196636. doi: 10.1371/journal.pone.0196636, PMID: 29995884 PMC6040685

[43] Miranda M Morici JF Zanoni MB Bekinschtein P. Brain-derived neurotrophic factor: a key molecule for memory in the healthy and the pathological brain. Front Cell Neurosci. (2019) 13:363. doi: 10.3389/fncel.2019.00363, PMID: 31440144 PMC6692714

[44] Verghese J. Cognitive and mobility profile of older social dancers. J Am Geriatr Soc. (2006) 54:1241–4. doi: 10.1111/j.1532-5415.2006.00808.x, PMID: 16913992 PMC1550765

[45] Beeri MS Leugrans SE Delbono O Bennett DA Buchman AS. Sarcopenia is associated with incident Alzheimer’s dementia, mild cognitive impairment, and cognitive decline. J Am Geriatr Soc. (2021) 69:1826–35. doi: 10.1111/jgs.17206, PMID: 33954985 PMC8286176

[46] Verghese J De Sanctis P Ayers E. Everyday function profiles in prodromal stages of MCI: prospective cohort study. Alzheimers Dement. (2022) 19:498–506. doi: 10.1002/alz.12681 35472732 PMC9596617

[47] Guzmán-García A Hughes JC James IA Rochester L. Dancing as a psychosocial intervention in care homes: a systematic review of the literature. Int J Geriatr Psychiatry. (2013) 28:914–24. doi: 10.1002/gps.3913, PMID: 23225749

[48] Vankova H Holmerova I Machacova K Volicer L Veleta P Celko AM. The effect of dance on depressive symptoms in nursing home residents. JAMDA. (2014) 15:582–7. doi: 10.1016/j.jamda.2014.04.013 24913212

[49] Wang Y Mandong L Tan Y Dong Z Wu J Cui H. Effectiveness of dance-based interventions on depression for persons with MCI and dementia: a systematic review and Meta-analysis. Front Psychol. (2022) 12:709208. doi: 10.3389/fpsyg.2021.709208, PMID: 35069306 PMC8767071

[50] Wei W Karim HT Lin C Mizuno A Andreescu C Karp JF. Trajectories in cerebral blood flow following antidepressant treatment in late-life depression: support for the vascular depression hypothesis. J Clin Psychiatry. (2018) 79:18m12106. doi: 10.4088/JCP.18m12106 PMC6419103 30358242

[51] Basso JC Satyal MK Rugh R. Dance on the brain: enhancing intra- and inter-brain synchrony. Front Hum Neurosci. (2021) 14:584312. doi: 10.3389/fnhum.2020.584312, PMID: 33505255 PMC7832346

[52] Gujing L Hui H Xin L Lirong Z Yutong Y Guofeng Y. Increased insular connectivity and enhanced empathic ability associated with dance/music training. Neural Plast. (2019) 2019:1–13. doi: 10.1155/2019/9693109 PMC6526550 31198419

[53] Ellingsen DM Isenburg K Jung C Lee J Gerber J Mawla I. Dynamic brain-to-brain concordance and behavioral mirroring as a mechanism of the patient-clinician interaction. Sci Adv. (2020) 6:eabc1304. doi: 10.1126/sciadv.abc1304 33087365 PMC7577722

[54] Lockwood PL. The anatomy of empathy: vicarious experience and disorders of social cognition. Behav Brain Res. (2016) 311:255–66. doi: 10.1016/j.bbr.2016.05.048, PMID: 27235714 PMC4942880

[55] McGarry LM Russo FA. Mirroring in dance/movement therapy: potential mechanisms behind empathy enhancement. Arts Psychother. (2011) 38:178. doi: 10.1016/j.aip.2011.04.005

[56] Avenanti A Bueti D Galati G Aglioti SM. Transcranial magnetic stimulation highlights the sensorimotor side of empathy for pain. Nat Neurosci. (2005) 8:955–60. doi: 10.1038/nn1481, PMID: 15937484

[57] Bastiaansen JA Thioux M Keysers C. Evidence for mirror systems in emotions. Philos Trans R Soc Lond Ser B Biol Sci. (2009) 364:2391–404. doi: 10.1098/rstb.2009.0058, PMID: 19620110 PMC2865077

[58] de Sousa AM Fisher B. Your Mind on Dance, with Dr. Emily Cross. [Audio Podcast Episode]. Minds Matter; (2023). Available at: https://open.spotify.com/episode/6FOcSAyI0jWfG3z0nacgc5?si=YMVM2BbNRuyGgKmOvXP07w (Accessed February 4, 2023).

[59] Sturm VE Datta S Roy ARK Sible IJ Kosik EL Veziris CR. Big smile, small self: awe walks promote prosocial positive emotions in older adults. Emotion. (2022) 22:1044–58. doi: 10.1037/emo0000876, PMID: 32955293 PMC8034841

[60] Tippett K.. Dacher Keltner: The Thrilling New Science of Awe, [Audio Podcast Episode], On Being, The On Being Project; (2023). Available at: https://onbeing.org/programs/dacher-keltner-the-thrilling-new-science-of-awe/ (Accessed February 4, 2023).

[61] Voss P Thomas ME Cisneros-Franco JM de Villers-Sidani É. Dynamic brains and the changing rules of neuroplasticity: implications for learning and recovery. Front Psychol. (2017) 8:1657. doi: 10.3389/fpsyg.2017.01657, PMID: 29085312 PMC5649212

[62] Kempermann G Fabel K Ehninger D Babu H Leal-Galicia P Garthe A. Why and how physical activity promotes experience-induced brain plasticity. Front Neurosci. (2010) 4:189. doi: 10.3389/fnins.2010.00189 21151782 PMC3000002

[63] Han Y Yuan M Guo YS Shen XY Gao ZK Bi X. The role of enriched environment in neural development and repair. Front Cell Neurosci. (2022) 16:890666. doi: 10.3389/fncel.2022.890666, PMID: 35936498 PMC9350910

[64] Queen NJ Hassan QN 2nd Cao L. Improvements to Healthspan through environmental enrichment and lifestyle interventions: where are we now? Front Neurosci. (2020) 14:605. doi: 10.3389/fnins.2020.00605, PMID: 32655354 PMC7325954

[65] Robertson IH. A noradrenergic theory of cognitive reserve: implications for Alzheimer’s disease. Neurobiol Aging. (2013) 34:298–308. doi: 10.1016/j.neurobiolaging.2012.05.019, PMID: 22743090

[66] Robertson IH. The stress test: can stress ever be beneficial? J Br Acad. (2017) 5:163–76. doi: 10.5871/jba/005.163

[67] Albright AC. Life practices In: Midgelow VL, editor. The Oxford Handbook of Improvisation in Dance. Oxford: Oxford University Press (2019)

[68] McDowall L. Exploring uncertainties of language in dance improvisation In: Midgelow VL, editor. The Oxford Handbook of Improvisation in Dance. Oxford: Oxford University Press (2019)

[69] Zwir I Del-Val C Hintsanen M Cloninger KM Romero-Zaliz R Mesa A. Evolution of genetic networks for human creativity. Mol Psychiatry. (2022) 27:354–76. doi: 10.1038/s41380-021-01097-y, PMID: 33879864 PMC8960414

[70] Kim SJ Kim SE Kim HE Han K Jeong B Kim JJ. Altered functional connectivity of the default mode network in Low-empathy subjects. Yonsei Med J. (2017) 58:1061–5. doi: 10.3349/ymj.2017.58.5.1061, PMID: 28792155 PMC5552636

[71] Beaty RE Benedek M Kaufman SB Silvia PJ. Default and executive network coupling supports creative idea production. Sci Rep. (2015) 5:10964. doi: 10.1038/srep10964, PMID: 26084037 PMC4472024

[72] Donnay GF Rankin SK Lopez-Gonzalez M Jiradejvong P Limb CJ. Neural substrates of interactive musical improvisation: an FMRI study of ‘trading fours’ in jazz. PLoS One. (2014) 9:e88665. doi: 10.1371/journal.pone.0088665, PMID: 24586366 PMC3929604

[73] Limb CJ Braun AR. Neural substrates of spontaneous musical performance: an FMRI study of jazz improvisation. PLoS One. (2008) 3:e1679. doi: 10.1371/journal.pone.0001679, PMID: 18301756 PMC2244806

[74] Ellis M Astell A. Adaptive Interaction and Dementia: How to Communicate without Speech. London: Jessica Kingsley Publishers (2018).

[75] Vigliotti AA Chinchilli VM George DR. Evaluating the benefits of the TimeSlips creative storytelling program for persons with varying degrees of dementia severity. Am J Alzheimers Dis Other Dement. (2019) 34:163–70. doi: 10.1177/1533317518802427, PMID: 30295037 PMC10852417

[76] Kaczmarska M. Stories in the Moment: Creating Shared Spaces of Belonging for and with People Living with Dementia. In Dance.; (2022). Available at: https://dancersgroup.org/2022/06/stories-in-the-moment/ (Accessed February 4, 2023).

[77] Lerman L. Hiking the Horizontal: Field Notes from a Choreographer. Middletown, Connecticut: Wesleyan University Press (2014).

[78] Tucker T. “Interview with Emily Cross”. George Mason University Arts Research Center; (2021). Available at: https://masonarc.gmu.edu/an-interview-with-emily-cross/ (Accessed February 4, 2023).

