# Supplementary material for: Valuing embodiment: insights from dance practice among people living with dementia
Source: Front Neurol. 2023 Jun 5;14:1174157. doi: 10.3389/fneur.2023.1174157 (PMC10277497; doi:10.3389/fneur.2023.1174157)
Supplement: Supplementary file 1 [file Data_Sheet_1.PDF]

Box 1 Case study of the value of a co-creative dance program for people living with dementia

Stories in the Moment™ is grounded on the understanding that dance is a universal language. We come to life with histories, with bodies that hold and were created through those histories and movement patterns inscribed by them.[68] Stories in the Moment™ amplifies participants' individual histories and reflections through collaboratively created dance stories. Co-creative dance extends self-hood and agency for people living with dementia in part through the process of mirroring. This is illustrated by reflections of a participant in Stories in the Moment™ on the use of dance mirroring with her husband (living with dementia at the time). "[He] has lost so much control over his life. And I hate that for him...when we do dancing in the kitchen, we'll do movements like you do with us…he'll start a movement and I will follow his movement…so he's in charge. And then he'll have this big smile on his face. Because, you know, he came up with the movement, and I'm doing the movement with him."[76]

Co-creative activities in Stories in the Moment™ expand upon the concept of mirroring to collaborate with people living with dementia to shape their ideas into miniature choreographed dances, enhancing their tools of embodied communication and recognizing their contribution as having value to the whole of the group. Care partners recognize how the approach of individualized attention extends a sense of wellbeing to participants. "Your inviting attention to each participant and the approach you use to put physical movement, music and storytelling together is exceptional. You draw in [their] attention, add a smile to [their] face and overall seem to greatly improve [their] mood." People living with dementia comment that the program "makes me feel better" and "makes me feel positive." (unpublished reflections from Stories in the Moment™ participants) This coupling of individual and collective creative collaboration promotes a sense of being seen and of having inherent value within the group and beyond. Stories in the Moment™ participants comment on this: "…he feels valued, which is super important to him."[76] This relationality and feeling of community and belonging persists from class to class. Too often for people living with dementia, access to this basic human right of relational citizenship is profoundly rare. Co-creative dance programs like Stories in the Moment™ offer fertile spaces to cultivate meaning and relationality adding value to and beyond the physiological benefits of dance on brain health.
